# Hospital Transfer Decision-Making for Residents with Dementia in Long-Term Care: A Qualitative Study of Interprofessional Perspectives

**DOI:** 10.3390/healthcare14203365

**Published:** 2026-10-09

**Authors:** Becky Tsarfati, Iris Manor Binyamini, Shiri Shinan-Altman

**Affiliations:** 1 Louis and Gabi Weisfeld School of Social Work, Bar Ilan University, Ramat-Gan 5290002, Israel; beckyt@edu.aac.ac.il (B.T.);; 2 Research Center for Innovation in Social Work, Faculty of Humanities and Social Sciences, Tel-Hai University of Kiryat Shmona in the Galilee, Kiryat Shmona 1220800, Israel

**Keywords:** dementia, long-term care, nursing homes, hospital transfer, interprofessional decision-making, continuity of care, resident safety, quality of care, clinical uncertainty

## Abstract

**Highlights:**

**What are the main findings?**

- Transfer decisions followed a staged process in which nurses first identified clinical changes, translated them into professional information, consulted within the interprofessional hierarchy, and physicians formally authorized hospital transfer.
- Limited access to on-site diagnostic and treatment resources shifted uncertain cases toward hospital referral, particularly in situations involving suspected fracture, head injury, DVT, respiratory deterioration, aspiration, or infection.

**What are the implications of the main findings?**

- Decision-support protocols should focus on recurrent high-uncertainty referral situations, including falls, suspected fracture, respiratory deterioration, suspected DVT, aspiration, and infection.
- Preserving continuity of care should be considered a resident-safety goal when deciding whether a resident with dementia can be monitored or treated safely within the long-term care facility.

**Abstract:**

**Background**: Hospital transfers of residents with dementia may provide them with access to acute diagnosis and treatment but also disrupt their continuity of care, which is essential for residents’ stability, safety, and quality of care. The aim of this study was to examine how transfer decisions are produced within the organizational context of long-term dementia care and how they may affect continuity of care. **Methods**: A qualitative descriptive design was used, supplemented by retrospective referral data. The study was conducted in a 200-bed dementia-specialist long-term care facility. Referral records over three years were reviewed to describe transfer patterns, reasons, and outcomes. Semi-structured interviews were conducted with 15 nurses and 2 physicians. Referral data were analyzed descriptively, and interviews were analyzed using Reflexive Thematic Analysis (RTA). **Results**: A total of 578 referrals to hospital emergency departments were identified; 34.9% resulted in hospitalization. Three themes were identified: (1) From recognition to authorization, describing interprofessional hierarchy in referral decision-making; (2) When uncertainty becomes risk, describing how limited diagnostic and treatment capacity shifted decisions toward transfer; and (3) Protecting continuity, describing staff concerns about the disruptive effects of transfer. Overall, decisions were shaped by clinical uncertainty, organizational conditions, and continuity-of-care concerns. **Conclusions**: Transfer decisions for residents with dementia are context-dependent organizational judgments, not solely responses to acute clinical deterioration. Strengthening institutional capacity, decision-support tools, staff training, and advance care planning may help preserve continuity of care, reduce avoidable disruption, and improve resident safety and quality of care.

## 1. Introduction

A substantial proportion of people living with dementia eventually require residential or nursing-home care as cognitive impairment, functional dependency, behavioral symptoms, and caregiving needs increase. A recent systematic review and meta-analysis found that approximately 13% of people with dementia were admitted to a nursing home within the first year after diagnosis, increasing to 57% within five years [1]. Residents in these settings often have complex care needs related to advanced age, multimorbidity, and polypharmacy, which increase the likelihood of their transfer to emergency departments and acute hospital care [1,2]. For residents with dementia, the decision to transfer them to a hospital involves more than access to acute diagnosis and treatment; it also requires careful consideration of continuity of care, as transferring them may disrupt familiar routines, staff knowledge of residents’ baseline condition, and stable care relationships, while exposing residents to delirium-related complications and cognitive, functional, and psychological decline [3,4,5,6].

Therefore, examining the decision-making process is essential to building a system that supports continuity of care. In dementia-specialist nursing homes, acute transfers are commonly triggered by suspected infection, falls with possible fracture or head injury, respiratory deterioration, or cardiovascular instability [3,4]. Previous studies suggest that some transfers may be potentially avoidable, depending on staff composition, organizational resources, and the facility’s ability to assess and manage acute changes on site [5,6,7].

A recent systematic review further suggested that variation in emergency department transfer rates may reflect potentially modified organizational and care-process factors within long-term care facilities [6].

Transfer decisions occur at a clinical–organizational junction shaped by medical, nursing, ethical, legal, and logistical considerations [5,6]. They are enacted through role-structured interprofessional processes in which nurses often identify acute changes and raise concerns, whereas physicians retain formal authority to authorize transfer [7]. In practice, these decisions are frequently made under conditions of uncertainty, particularly when staff must balance the potential benefits of hospital assessment and treatment against the risks of transfer-related harm and disruption to continuity of care [1,8]. Intervention-focused evidence suggests that reducing emergency department attendances and hospital admissions from long-term care requires multifaceted strategies, including advance care planning, shared decision-making, interdisciplinary teamwork, improved communication, timely clinical support, and stronger capacity for assessment and treatment within facilities [9].

Although the literature on nursing-home transfers is substantial, previous studies have focused mainly on transfer rates, contributing factors, appropriateness, potentially avoidable transfers, and interventions to reduce unnecessary hospital use [4,7,8,9,10]. Less is known about how transfer decisions are made in real time within long-term care settings, and how interprofessional roles, institutional capacity, family involvement, accountability concerns, and continuity-of-care considerations combine to shape the final decision. In the Israeli long-term care context, hospital transfer decisions are shaped not only by residents’ clinical condition, but also by the organization of care within the facility, physician availability, access to diagnostic and treatment resources, family and surrogate involvement, and the availability of advance care preferences. These contextual factors are especially important in dementia-specialist facilities, where residents may be unable to communicate symptoms clearly and where acute changes must often be interpreted through staff familiarity with the baseline condition. In this study, continuity of care is understood as a multidimensional construct that includes informational continuity, management continuity, and relational continuity. Informational continuity refers to the use of knowledge about the resident’s history, baseline function, preferences, and current condition. Management continuity refers to coherent and coordinated care across professionals, shifts, and settings. Relational continuity refers to ongoing therapeutic relationships between residents, families, and familiar staff. For residents with dementia, hospital transfer may disrupt all three dimensions by separating the residents from familiar caregivers, routines, clinical knowledge, and individualized care practices [11,12].

This study aimed to examine how hospital transfer decisions for residents with dementia are produced within the organizational context of long-term care. Specifically, it explored how clinical uncertainty, interprofessional roles, institutional resources, family involvement, medico-legal concerns, and continuity-of-care considerations shape decisions to transfer residents to the hospital or manage them within the facility. The findings position hospital transfers not as isolated clinical responses, but as organizationally shaped judgments influenced by clinical, institutional, interprofessional, family, and continuity-of-care considerations. The contribution of this study lies not in identifying hospital-transfer factors in isolation, many of which have been described previously, but in showing how these factors are assembled in practice within a dementia-specialist long-term care facility in Israel. By linking staff accounts with three years of referral data, the study offers a context-specific account of hospital transfer decision-making and reframes continuity of care as a resident-safety consideration in dementia long-term care.

## 2. Materials and Methods

### 2.1. Design

A qualitative descriptive design was employed to explore interprofessional decision-making regarding the transfer of residents with dementia from long-term care to hospital emergency departments. Retrospective referral data were used to describe the scope, reasons, timing, ward type, and outcomes of transfers over a three-year period and to provide structured context for interpreting the interview findings. This design was selected because the study aimed to examine staff accounts, professional reasoning, and organizational processes related to hospital transfer decision-making in dementia long-term care [13].

### 2.2. Setting

The study was conducted in a 200-bed, six-unit dementia-specialist long-term care facility in Israel. Units were organized according to residents’ functional and ambulatory status, including wards for ambulatory residents with dementia and wards for fully dependent, non-ambulatory residents with dementia. Nursing staff were present across shifts and were responsible for ongoing monitoring, identification of acute changes, and communication with physicians. Physicians were physically present on site during routine daytime working hours. Outside these hours, including late afternoon, evening, night, weekends, and holidays, medical consultation was provided by an on-call physician, usually by telephone. The facility provided in-house treatments only within the scope approved by Ministry of Health regulations, physician orders, and institutional protocols. These included selected treatments such as oxygen therapy, antibiotics, subcutaneous fluids, and other approved medications when clinically indicated. Participants emphasized that treatments outside the approved regulatory or institutional scope, including certain opioid or palliative medication regimens, could not be initiated independently within the facility and required appropriate medical authorization, prescribing arrangements, or hospital-based evaluation. The facility did not have Ministry of Health authorization to administer intravenous medications, and advanced diagnostic resources, including imaging, CT, Doppler ultrasound, and comprehensive laboratory testing, were not available on site. Consequently, suspected fracture, head injury, Deep Vein Thrombosis (DVT), respiratory deterioration, gastrointestinal bleeding, altered consciousness, hemodynamic instability, or unexplained acute decline often required hospital referral for diagnostic clarification, intravenous treatment when needed, and risk management.

### 2.3. Participants and Sampling

Participants were recruited using purposive sampling to ensure variation across professional roles, seniority, ward affiliation, and involvement in hospital transfer decision-making [14]. Eligible participants were nursing and medical staff members who were directly involved in identifying, assessing, authorizing, or managing transfers of residents with dementia from the facility to hospital emergency departments. Potential participants were approached by the principal investigator, received written and verbal information about the study, and were invited to participate voluntarily. Seventeen staff members agreed to participate, including 15 nursing staff members and two physicians. Participants represented both managerial and frontline roles across the facility. Written informed consent was obtained from participants and was verbally confirmed before the interviews began. Participant characteristics are presented in Table 1.

### 2.4. Data Collection Procedure

Ethical approval was obtained from the local institutional review board of the dementia-specialist long-term care facility on 29 September 2025 (AUD-ALZ-010). Confidentiality was maintained through transcript de-identification and reporting findings in aggregate form. For the retrospective review of residents’ referral records, the requirement for individual informed consent was waived by the institutional review board because the analysis used existing clinical documentation and de-identified data.

Data collection consisted of two components. First, a retrospective review of all referral events documented in residents’ records from 1 September 2022, to 1 September 2025, was conducted. The unit of analysis was the referral event rather than the individual resident; therefore, repeated referrals involving the same resident were included and interpreted as clustered observations rather than statistically independent cases. A referral event was defined as a documented decision or action to refer a resident from the long-term care facility to a hospital emergency department for acute assessment or treatment. Hospitalization was defined as admission to a hospital ward after emergency department assessment, whereas non-hospitalization referred to emergency department assessment followed by return to the facility. Family refusal was defined as a documented case in which hospital referral was recommended or initiated but did not proceed because the family or legal proxy refused transfer.

Data were extracted from mandatory documentation in residents’ records by the principal investigator, a registered nurse familiar with institutional terminology and documentation practices. Extracted variables included date and time of referral, documented referral reason, primary and additional diagnoses, ward type, and referral outcome. Cases initially documented as “Other” were reviewed using the available referral indication and diagnosis fields. When a more specific clinical indication could be identified, the referral event was reclassified into the relevant clinically meaningful category; otherwise, it remained in the residual “Other causes” category.

Second, semi-structured interviews were conducted between December 2025 and January 2026 with nursing and medical staff. Interviews were conducted by the principal investigator, a PhD senior nursing academic who was not employed by the study facility and had no supervisory relationship with participants. Interviews were conducted face-to-face or via secure videoconference, according to participant preference, and lasted approximately 45–60 min. All interviews were conducted in Hebrew in the presence of only the participant and the interviewer. They were audio-recorded with participants’ consent, transcribed verbatim, and accompanied by reflexive field notes. All 17 interviews were included in the analysis. The final sample size was determined pragmatically and analytically, reflecting the available pool of eligible staff who were directly involved in hospital transfer decision-making during the study period, as well as the relevance, specificity, depth, and variation in the interview accounts in relation to the study aim.

### 2.5. Research Instruments

Two data sources and corresponding research tools were used. First, a structured data extraction form was used to document referral characteristics, reasons for transfer, ward type, and referral outcomes from retrospective referral records. Second, a semi-structured interview guide was used to elicit participants’ involvement in transfer decisions, the workflow and authorization process, case-based reflections on real-world transfer decisions, alternatives to transfer, and the resolution of disagreement within the team.

Semi-structured interviews were selected because the study aimed to explore how staff members interpret and explain hospital transfer decisions in real clinical practice. This method was appropriate for examining professional reasoning, clinical uncertainty, interprofessional roles, family involvement, institutional constraints, and continuity-of-care considerations. It also enabled participants to describe concrete cases and decision-making processes in their own words, while ensuring that core topics were addressed consistently across interviews. Alternative methods, such as structured questionnaires or retrospective record review alone, would have provided less insight into the contextual judgments, professional reasoning, and interprofessional dynamics underlying transfer decisions.

Example interview questions included: “To what extent are you involved in decisions regarding the transfer of residents?”, “In what medical situations do you think a transfer to a hospital should be considered?”, and “Can you describe how the decision-making process for a transfer develops, including who initiates it, who participates, and who makes the final decision?”.

### 2.6. Data Analysis

Retrospective referral-record data were summarized using descriptive statistics to characterize the scope and profile of hospital transfer decisions. The unit of analysis was the referral event rather than the individual resident. Frequencies and percentages were calculated overall and stratified by ward type and referral outcome. Referral events were also summarized descriptively by year, time of day, and destination hospital to provide additional contextual information.

Referral reasons were grouped into clinically related categories and tabulated to identify the most common documented clinical triggers within each ward type. Referral-reason categories were classified using the original referral-reason field, the more detailed chart-based referral indication, and the documented diagnosis when available. In cases involving multiple indications, the primary documented reason for referral was used for classification, while secondary indications were considered when assigning the case to a clinically related category. Cases initially documented as “Other” were reviewed to retrieve the most specific available clinical indication. When the available documentation supported reassignment to a clinically meaningful category, the referral event was reclassified accordingly. When the documentation remained heterogeneous, infrequent, or insufficiently specific to support a separate stable category, the referral event was retained within the residual “Other causes” category.

Because repeated referrals involving the same resident could occur, referral events were treated as clustered observations and were interpreted descriptively rather than as statistically independent cases. No inferential comparisons between ward types were conducted.

Interview transcripts were analyzed using Reflexive Thematic Analysis (RTA), guided by Braun and Clarke’s six-phase framework and contemporary methodological guidance on reflexive thematic analysis [15,16,17,18]. The analysis was primarily inductive and semantic, focusing on participants’ accounts of how hospital transfer decisions were understood, justified, and enacted in practice, while also attending to broader organizational meanings related to uncertainty, risk, professional responsibility, and continuity of care. All 17 interviews were included in the analysis, and no interviews were excluded after data collection. The study does not claim thematic saturation. Instead, sample adequacy was understood in terms of information power, reflecting the relevance of participants’ roles to the study aim, the specificity of the participant group, and the depth and variation in the interview accounts [17,19].

The principal investigator led the initial familiarization, coding, and preliminary theme development through repeated reading of the transcripts and generation of initial codes related to clinical change, referral decision-making, interprofessional roles, institutional capacity, family involvement, and continuity-of-care considerations. Candidate themes were then discussed within the research team, reviewed against the coded extracts and the full dataset, and refined to improve coherence, distinctiveness, and relevance to the research aim. Themes were defined and named to reflect the central organizing concepts identified in the data and were developed into an integrated analytic account of how hospital transfer decisions were produced within the organizational context of dementia long-term care. The analysis focused on patterned meanings across staff accounts rather than quantified frequencies of participant responses. No qualitative data-analysis software was used; transcripts, codes, and analytic notes were managed manually.

Throughout the analysis, attention was given to variation by professional role and to the relationship between interview narratives and the descriptive referral data. The retrospective referral data were not used to generate, confirm, or validate themes. Rather, integration occurred during interpretation by considering descriptive referral patterns alongside the qualitative themes to contextualize staff accounts.

#### 2.6.1. Translation Procedure

All interviews were conducted, audio-recorded, transcribed, and analyzed in Hebrew. Theme development and analytic discussions were also conducted in Hebrew to preserve participants’ original meanings and professional terminology. After the themes were finalized, selected illustrative quotations were translated into English for publication. The translated quotations were reviewed by bilingual members of the research team to ensure semantic accuracy, preservation of clinical and organizational meaning, and consistency with the original Hebrew transcripts.

#### 2.6.2. Trustworthiness and Reflexivity

Trustworthiness was supported through reflexive engagement with the data, verbatim transcription, transparent documentation of analytic decisions, team discussions, and comparison of interview accounts with referral-record patterns for contextual interpretation. Interviews were audio-recorded and transcribed verbatim to preserve participants’ accounts and allow repeated engagement with the source material. The use of a semi-structured interview guide ensured that core topics were addressed across interviews while allowing participants to provide detailed case-based descriptions. Reflexive notetaking supported ongoing awareness of how the researchers’ professional backgrounds might shape interpretation. The three-year referral records were used to contextualize the interview findings and to provide background regarding the scope, timing, reasons, and outcomes of referrals, rather than as a source of independent validation.

In accordance with Reflexive Thematic Analysis principles, reflexivity was embedded throughout the process of theme development. The principal investigator is a PhD senior nursing academic and head of an undergraduate nursing program with more than 35 years of professional experience. She had no employment or supervisory relationship with the study site but had longstanding familiarity with gerontological nursing and interprofessional teamwork through teaching and clinical supervision. Repeated engagement with the dataset, reflexive notetaking, and team discussions were used to monitor how prior knowledge in patient safety, gerontological nursing, and risk management might shape interpretation and to ensure that themes remained grounded in participants’ narratives [13].

This study was reported in accordance with the Consolidated Criteria for Reporting Qualitative Research (COREQ). The complete COREQ checklist is provided as Supplementary File S1.

## 3. Results

### 3.1. Descriptive Findings: Reasons for Referral to Hospital Emergency Departments

A retrospective review of residents’ records identified 578 referral events from the dementia-specialist long-term care facility to hospital emergency departments between 1 September 2022, and 1 September 2025. These referral events involved 246 unique residents, indicating that some residents contributed more than one referral event during the study period. The unit of analysis was the referral event rather than the individual resident. Repeated referrals involving the same resident were included; therefore, referral events were clustered within residents and are reported descriptively. The crude referral-event rate was 0.96 referral events per bed-year over the three-year study period, calculated as 578 referral events divided by 200 beds and three years. This estimate should be interpreted cautiously because it does not account for occupancy rates or repeated referrals involving the same resident.

Of the 578 referral events, 419 (72.5%) originated from wards caring for fully dependent, non-ambulatory residents with dementia, and 159 (27.5%) originated from wards caring for ambulatory residents with dementia (Table 2). Overall, 202 of 578 referral events (34.9%) resulted in hospitalization, 370 of 578 referral events (64.0%) did not result in hospitalization, and in 6 of 578 cases (1.0%) the family refused transfer. Among fully dependent, non-ambulatory residents with dementia, 154 of 419 referral events (36.8%) resulted in hospitalization. Among ambulatory residents with dementia, 48 of 159 referral events (30.2%) resulted in hospitalization. Family refusal was documented in 3 of 419 referral events (0.7%) among fully dependent, non-ambulatory residents and 3 of 159 referral events (1.9%) among ambulatory residents (Table 2).

Referral reasons were grouped into clinically related categories. Among ambulatory residents with dementia, the most frequent referral category was fall, trauma, head injury, or suspected fracture (50/159; 31.4%), followed by respiratory deterioration, pneumonia, or aspiration (23/159; 14.5%). Among fully dependent, non-ambulatory residents with dementia, the most frequent referral category was respiratory deterioration, pneumonia, or aspiration (95/419; 22.7%), followed by fall, trauma, or head injury, or suspected fracture (43/419; 10.3%). The full distribution of referral reason categories by ward type is presented in Table 3.

Initially, 161 referral events were documented as “Other.” After reviewing the available referral indication and diagnosis fields, 21 events were reclassified into clinically meaningful categories when a more specific clinical indication could be identified. The remaining 140 events were retained in the residual “Other causes” category because the documentation was heterogeneous, infrequent, or insufficiently specific to support a separate stable category. This category should therefore be interpreted as a limitation of retrospective referral classification rather than as missing referral documentation.

Referral events were also examined by year, time of day, and destination hospital to provide contextual information for interpreting the decision-making process. Across the study period, 37 of 578 referral events (6.4%) occurred in 2022, 194 (33.6%) in 2023, 200 (34.6%) in 2024, and 147 (25.4%) in 2025. Because the study period extended from 1 September 2022, to 1 September 2025, data from 2022 and 2025 represent partial calendar years and are therefore presented descriptively.

Referral timing was examined descriptively. Most referral events occurred during the morning or early afternoon shift (07:00–14:59; 372/578, 64.4%), while 152 referrals (26.3%) occurred during the late afternoon or evening shift (15:00–22:59), and 54 referrals (9.3%) occurred during the night or early morning shift (23:00–06:59). Overall, 206 referrals (35.6%) occurred after 15:00. These data are presented descriptively and were not used to infer differences in referral decision-making by shift. Most referral events were directed to Sheba Medical Center (538/578, 93.1%), reflecting the facility’s routine referral pathway and geographic-service context.

### 3.2. Qualitative Findings

The thematic analysis identified three central themes and two subthemes per theme describing how staff made decisions regarding the transfer of residents with dementia to acute-care settings (Table 4). Together, the themes portray referral decision-making as a staged process: first, clinical information was identified and translated within a structured professional hierarchy; second, staff made decisions under structural and operational constraints that could shift decisions toward transfer, even without a definitive clinical need; and third, the team weighed the expected medical benefit of transfer against its potential harm to continuity of care and resident safety. Although these dimensions were closely related in practice, they were treated as analytically distinct: the first theme focuses on the formal structure of decision-making and authorization, the second on how clinical uncertainty and institutional capacity shaped risk management, and the third on how continuity of care was weighed as a resident-safety consideration.

#### 3.2.1. Theme 1. From Recognition to Authorization: Interprofessional Hierarchy in Referral Decision-Making

This theme presents the starting point of the referral process: a structured division of roles in which nurses identify acute deterioration, assess deviations from the resident’s baseline condition, and translate these changes into clinically actionable information for physician consultation. Participants’ accounts positioned nursing assessment as the basis for medical authorization, while physicians retained formal authority to approve transfer out of the facility.

“*First, the nurse is the one who identifies the problem; she is the one who takes the initial indicators and does an initial screening of what is happening and reports to the doctor. If the doctor is in the ward, he will come to check; if not, it will be by phone. If the nurse is alone, that is, if there is no doctor in the building, then many times they will consult with each other as if another eye or two will come to see what is happening and express their opinion. They will talk on the phone with my deputy or me. And many times, if they are alone, they will tell the doctor, ‘I think the resident should be sent,’ and the doctor will understand and accept that, and then the resident will be sent.*”

(PN 3)

Subtheme 1. Initial nursing assessment and identification of clinical change: Nurses’ continuous presence on the ward and familiarity with residents’ baseline functioning positions them as key identifiers of early clinical change. Their assessments include vital signs as well as behavioral, functional, and communicative cues, which are reported to physicians as part of the referral decision-making process.

“*Look at our advantage that we know the residents and their basic situation, meaning we can pretty quickly recognize that something is happening. The problem with people with dementia is that they won’t tell you, ‘I’m in pain, I’m not feeling well, something is happening to me.’ You can’t get feedback from them; you need to know and get to know them, and because they live here, we know them and know the baseline, so it’s easy to see who’s out of balance. Someone who is quieter and we see that they haven’t passed stool for, for example, two days, we’ll do the intervention without them complaining, and we need to see that the treatment is really helping. So, the fact that we know them is a very significant part in deciding whether to transfer or not.*”

(PN3)

Subtheme 2. Hierarchy of authority and professional safety nets: Although physicians hold the formal authority to approve transfer, participants described nurses’ assessments as central to the decision-making process, especially during evening and night shifts when physicians are not physically present. In these situations, participants described consulting with colleagues, senior nurses, nursing managers, and physicians.

“*For every transfer to the hospital, we send a message in our nurses’ WhatsApp group indicating who was transferred, why they were transferred, and who accompanied them, so that is how I stay updated…. This is something that exists in all departments. It is very helpful and makes the work much easier. We also update the group only about exceptional events …. I can contact the institution’s WhatsApp group to consult with other nurses or the institution’s nursing manager…. I feel more confident in deciding whether to report a change in the resident’s condition to the doctor after consulting with other nurses when I am uncertain.*”

(PN5)

#### 3.2.2. Theme 2. When Uncertainty Becomes Risk: Clinical Uncertainty, Limited Resources, and Defensive Transfer

This theme captures how clinical uncertainty became a driver of hospital transfer when serious conditions could not be confidently confirmed, excluded, or managed within the facility. Staff accounts suggested that transfer was often considered not only because deterioration had already been confirmed, but because limited on-site diagnostic capacity and restricted treatment options made hospital referral the safer course of action under conditions of incomplete information, time pressure, and perceived accountability.

“*First, it’s a shame that there is no approval to give medications like narcotic therapy to relieve symptoms for terminally ill people. If, say, I had morphine in an institution that I could give to relieve the pain, then that would be an excellent tool, but I don’t have that. That’s why I send patients who are in the final stages of their lives and are suffering, and it’s a shame because I think they would prefer to die in their ward, under their own conditions, in their own home, in an environment they know, with the staff they know. That’s unequivocally true. If I really had blood tests available 24/7, then I could also give some kind of treatment, like electrolyte disorders, for example.*”

(PN13)

Subtheme 1. Decision-making under lack of information and resource constraints: participants described hospital referral as necessary in situations involving acute deterioration or diagnostic uncertainty, particularly when the facility lacked the resources to confirm or exclude serious conditions on site.

“*The facility does not have the capacity to perform X-rays or CT scans. Therefore, every case of a fall with suspected fracture or head injury in a patient receiving anticoagulants requires immediate referral to the emergency department for imaging, since there is no other way to rule out intracranial bleeding or fracture.*”

(PN 7)

*“Yesterday, a resident woke up in the morning with a swollen leg and pain, and there was a suspicion of deep vein thrombosis in the leg. Because we couldn’t test it here, we had to refer him to the emergency room.*”

(PN 10)

“*Our institution does not have the tools and resources to make the diagnosis; it is not possible to do an X-ray… it is not possible to do a CT scan…. If I had lab tests and saw that he did not have high leukocytes, I would not refer him.*”

(PN 14)

Subtheme 2. Hospital transfer as defensive and systemic risk management: Participants’ accounts described hospital referral in relation to family expectations and concerns about professional accountability. These concerns were especially apparent in situations in which the resident’s condition was unclear, or family members questioned the decision not to transfer.

“*I remember once a resident in terminal condition with a family request not to send her [to the hospital]. We hooked her up to oxygen, and I called the doctor, who also said he knew the family didn’t want to send her [to the hospital]. But the son called and said, ‘Why aren’t you sending her?’ and he was very stressed, and we sent her. She died in the emergency room, and her son was very angry and said he hadn’t given an order not to send her to the emergency room, and it was his word against mine. Since that case, I always send people to the emergency room because that’s the only way I feel protected.*”

(PN 5)

“*Failure to refer a resident who needs clarification could be considered an ‘exceptional event’ if harm is caused, so the referral serves as a protective tool for the staff and the institution.*”

(PN 14)

#### 3.2.3. Theme 3: Protecting Continuity: Balancing Hospital Transfer Against the Harms of Disruption

This theme presents continuity of care as a clinical and safety consideration in decisions about hospital transfer. Participants’ accounts positioned the familiar institutional environment, daily routines, and ongoing relationships with staff as important resources for maintaining residents’ cognitive, emotional, and functional stability. Hospital transfer was therefore described as a double-edged decision: it could provide access to urgent medical assessments, but it could also disrupt familiar care, increase confusion and dis-tress, and complicate recovery after return to the facility.

Subtheme 1. Risk versus benefit: Transfer-related harms for residents with dementia. Participants’ accounts emphasized that the familiar institutional environment, daily routine, and long-standing relationships with staff were central to maintaining residents’ cognitive, emotional, and functional stability.

“*The best treatment for Alzheimer’s is routine. Any change can trigger events of disease progression.*”

(PN 2)

“*There was a case of a resident who came back from hospitalization and didn’t even recognize the staff he knew. Before the hospitalization he even recognized me by name. He went to the hospital for a week, had a pelvic fracture, had some surgery, and came back to us, didn’t recognize anyone, and was restless. At the hospital they couldn’t give him any medicine; they couldn’t even when he refused to eat, so he needed more than a week for rehabilitation, but they couldn’t. The hospital ward told us that we had to return him to our ward. It continued for another week, and then he slowly came back to himself. We didn’t change anything in his medication, as if we gave him more sedatives, we would lose him.*”

(PN 15)

Hospital referrals were viewed as a disruption to the residents’ familiar environment and as a potential source of harm. Participants associated the transfer with the onset of confusion, agitation, delirium, infections, pressure injuries, functional and cognitive decline, and difficulty adjusting after returning.

“*Hospitalization causes both mental and functional deterioration. It also disrupts the residents’ entire support system. Hospitalization is not good in any way. Our goal, despite all the therapeutic difficulties, is to keep him here and not send him away. We know how much hospitalization lowers a person’s cognitive function. Rehabilitation afterwards to return to baseline is very difficult, very, very difficult, almost impossible.*”

(PN 11)

Subtheme 2. Professional experience and local management: Professional experience shaped participants’ accounts of local monitoring and treatment options before referral. More experienced staff described greater confidence in interpreting changes in relation to residents’ baseline condition and in reporting to physicians when necessary.

“*Because of my experience, I can monitor the resident’s condition and report to the doctor when necessary. I believe that we don’t always have to transfer them to a hospital….*”

“*To decide to transfer, you need to know how to accept responsibility, you need to know the residents. I know what is changing with the resident because and when I monitor his condition I can decide to report to the doctor if it is necessary.*”

(PN17)

Participants also referred to the need for structured decision support, particularly when decisions were uncertain.

“*It is better to have clear written instructions when a referral is needed. Let’s tentatively take a situation where someone needs to be referred now. I measure the symptoms, decide he should be referred, go to the doctor, and neither the doctor nor I are 100% sure it is necessary, but the doctor says, ‘Let’s refer.’ I am a professional, and if I know that this is a situation that can continue to be treated here, I will insist that he not be referred. If instructions can guide me, it would be very helpful.*”

(PN 12)

Overall, participants described referral decisions as involving clinical assessment, institutional resources, professional experience, family expectations, and continuity-of-care considerations.

## 4. Discussion

The main finding of this study is that hospital transfer decisions for residents with dementia in long-term care are not determined by clinical deterioration alone but are produced within a complex clinical–organizational context. Documented referral characteristics showed what prompted transfers were, whereas participants’ accounts explained how clinical, organizational, professional, family, medico-legal, and continuity-of-care considerations shaped transfer decisions. Integrating descriptive referral data with qualitative themes showed that hospital transfer decisions in dementia long-term care are shaped by residents’ acute clinical condition, institutional capacity, professional responsibility, family expectations, and continuity-of-care concerns.

This study extends previously transfer literature by moving beyond a list of factors associated with hospital referrals and showing how these factors operated together in real-time decision-making. In this setting, hospital transfer functioned as a form of risk work [20]: staff weighed clinical uncertainty, limited institutional capacity, professional accountability, family expectations, and potential disruption to continuity of care when deciding whether hospital assessment was necessary or whether the resident could be safely managed within the facility.

The retrospective referral data showed that 202 of 578 referral events (34.9%) resulted in hospitalization, whereas 370 of 578 referral events (64.0%) did not. This pattern should be interpreted cautiously. Non-hospitalization does not necessarily indicate that a referral was unnecessary, avoidable, or inappropriate. Rather, discharge from the emergency department may reflect diagnostic rule-out, treatment delivered in the emergency department, clinical stabilization or resolution, family preference, or the need for hospital-based assessment under conditions of uncertainty. This interpretation is consistent with the qualitative findings, which showed that transfer decisions were often made when the facility could not safely confirm, exclude, or manage potentially serious conditions on site. The findings refine existing knowledge about interprofessional decision-making in nursing-home transfers. Previous studies have emphasized communication gaps between nurses, physicians, residents, and families as contributors to transfer decisions [8,21,22,23]. In contrast, the present study found that interprofessional communication was frequent and embedded in routine practice. Nurses often identified acute changes, assessed deviations from the resident’s baseline condition, communicated clinical concerns, and initiated consultation, whereas physicians retained formal authority to authorize transfer. Thus, the central challenge was not merely lack of communication, but the limited ability of communication to compensate for unavailable diagnostic tools, restricted treatment options, and differences in professional confidence. This distinction shifts attention from communication alone to the organizational conditions that determine whether residents can be safely assessed and managed within the facility.

Professional experience emerged in participants’ accounts as an important factor in managing uncertainty. Experienced staff were described as more willing to monitor residents within the facility when they could interpret clinical changes in relation to the residents’ baseline condition, whereas less experienced staff were perceived as more likely to seek hospital transfer as a risk-reducing strategy. This finding reflects staff perceptions rather than a demonstrated behavioral difference. This finding extends previous research showing that staff judgment, risk perception, professional confidence, and organizational context shape nursing-home transfer decisions [20,21,22,23,24]. These accounts suggest that staff perceived experiential knowledge, professional confidence, and team support as important influences on how uncertainty was managed during transfer decision-making.

Referral timing provided additional context for interpreting the qualitative findings. Most referral events occurred during morning or early afternoon hours, while more than one-third occurred after 15:00. This descriptive pattern does not establish differences in decision-making by shift, but it helps contextualize participants’ accounts of evening and night decision-making, particularly situations in which nursing staff identify acute changes, consult physicians remotely, and initiate escalation when medical presence on site is more limited. These findings reinforce the need for clear pre-transfer assessment protocols and structured escalation pathways across all shifts.

Institutional capacity was another major factor shaping referral decisions. Participants described situations in which hospital transfer was considered necessary not because a serious condition had already been confirmed, but because the facility could not safely confirm or exclude it. This was particularly evident in suspected fracture or head injury, respiratory deterioration, suspected aspiration, suspected DVT, infection, gastrointestinal bleeding, cardiovascular instability, and altered consciousness. In these situations, the emergency department functioned as a site of diagnostic clarification and risk management, particularly when potentially serious conditions could not be safely confirmed or excluded within the facility. Instead, such transfers may reflect the limits of on-site assessment and treatment capacity rather than inappropriate professional judgment. This interpretation is consistent with literature showing that transfer decisions are shaped by diagnostic uncertainty, facility resources, staff confidence, and the availability of clinical support [5,6,7,14,18,21,24].

The role of family members added another layer of complexity, although this finding should be interpreted as reflecting staff perceptions rather than directly reported family perspectives. Although documented family refusal of transfer was rare, staff accounts suggested that families influenced real-time decision-making through perceived expectations, anticipated disagreement, emotional pressure, and medico-legal accountability. In these accounts, family involvement appeared to operate less through formal approval or refusal and more through how staff anticipated and managed family responses during uncertain clinical situations. Pulst et al. showed that relatives and legal guardians are often involved in decisions regarding unplanned hospital transfers from nursing homes [22]. Givens et al. further demonstrated that decisions about hospital transfer in advanced dementia are closely linked to goals of care and surrogate decision-making [20]. In the present study, family involvement therefore functioned as a staff-perceived relational and organizational factor that shaped how staff assessed risk under uncertainty.

A central contribution of this study is the positioning of continuity of care as a core dimension of resident safety in dementia long-term care. Continuity of care is commonly understood as including informational, management, and relational continuity [11]. These dimensions are particularly relevant during transitions involving older residents of long-term care facilities, where effective transitional care depends on communication between health-care professionals, timely and complete exchange of information, care planning, and preparation of residents and caregivers for transitions between settings [25]. The findings of the present study suggest that hospital transfer may disrupt all three dimensions. Informational continuity may be disrupted when hospital teams lack detailed knowledge of the resident’s baseline function, usual behavior, communication patterns, medication routines, and individualized care needs. Management continuity may be affected when care plans, monitoring practices, medication routines, and clinical priorities shift between the long-term care facility and the hospital. Relational continuity may be disrupted when residents with dementia are separated from familiar staff, routines, and relationships that support orientation, emotional security, and behavioral stability. In this sense, continuity of care functioned not only as a preference for familiar care, but also as a resident-safety consideration in transfer decision-making. The retrospective referral data showed that only about one-third of referrals resulted in hospitalization; however, non-hospitalization may reflect multiple clinical scenarios and should not be interpreted as indicating that transfer was unnecessary or avoidable. The interview findings reinforced this interpretation: staff described routine, familiarity, stable relationships, and knowledge of residents’ baseline functioning as protective clinical resources. Participants also emphasized that hospital transfer could disrupt these resources and expose residents to confusion, agitation, delirium, functional decline, cognitive deterioration, infection, pressure injuries, and difficulty readjusting after return to the facility. This finding is consistent with previous evidence showing poorer hospital outcomes among people with dementia and older adults with cognitive impairment, including delirium, functional decline, longer hospital stays, and increased care needs after discharge [23,26]. The present study extends this literature by showing that continuity of care should not be viewed only as a preferred feature of dementia care, but also as a resident-safety consideration that should be actively weighed during transfer decision-making.

The findings have direct implications for clinical practice and organizational policy. Reducing potentially avoidable transfers requires strengthening the capacity of long-term care facilities to manage acute changes when clinically appropriate. Intervention-focused evidence suggests that reducing emergency department attendances and hospital admissions from long-term care facilities requires multifaceted strategies rather than a single intervention [27]. In line with this evidence, the present findings support the need for structured pre-transfer assessment, clear decision-support protocols, timely medical consultation, improved access to diagnostic and treatment options, and targeted training for less experienced staff. Protocols should focus on recurrent high-uncertainty situations identified in the referral data, including falls and suspected fracture, head injury, respiratory deterioration, suspected aspiration, suspected DVT, infection, altered consciousness, and gastrointestinal bleeding.

The findings also support the need for stronger family communication and advance care planning. Families and legal guardians should be engaged before acute deterioration occurs to clarify goals of care, preferences regarding hospitalization, expectations regarding comfort-focused care, and circumstances in which treatment within the facility may be appropriate. Such discussions may reduce decision-making under pressure and support staff when balancing clinical risk, family expectations, continuity of care, and medico-legal accountability. In addition, structured post-transfer procedures are needed for residents who return from the hospital, including medication reconciliation, reassessment of cognitive and functional status, review of hospital recommendations, prevention of delirium and pressure injuries, and rapid re-establishment of familiar routines.

Overall, this study shows that hospital transfer decisions for residents with dementia are shaped by both clinical need and the organizational conditions in which care is delivered. Improving transfer decision-making requires strengthening institutional capacity to identify which situations require acute-care escalation and which can be safely managed within the familiar long-term care environment. Such support may enhance care quality and resident safety by preserving continuity of care, reducing avoidable disruption, and supporting more consistent interprofessional decision-making.

### 4.1. Practice Implications

The findings point to concrete clinical, organizational, and policy actions that may improve hospital transfer decision-making in dementia long-term care, although the effectiveness of specific interventions requires prospective evaluation. First, facilities should implement structured pre-transfer assessment protocols for recurrent high-uncertainty situations identified in the referral data, including falls and suspected fracture, head injury, respiratory deterioration, suspected aspiration, suspected DVT, infection, altered consciousness, gastrointestinal bleeding, and hemodynamic instability. These protocols should clarify which clinical signs require urgent hospital referral, which situations may be monitored safely within the facility, and when physician consultation is required.

Second, decision-making capacity within the facility should be strengthened across all shifts. This includes timely physician consultation, clear escalation pathways for evening and night decisions, and targeted support for less experienced nursing staff. Case-based training, simulation of common referral scenarios, and structured consultation with senior nurses and physicians may help reduce uncertainty and support more consistent transfer decisions.

Third, organizational leaders should examine whether selected diagnostic and treatment capacities can be improved within the boundaries of regulation and institutional policy. This may include improved access to point-of-care laboratory testing, urgent imaging pathways or mobile radiography, and clearer arrangements for hospital-based diagnostic clarification when on-site assessment is insufficient. Where regulatory restrictions limit selected treatments, such as intravenous medication administration, facilities may consider consultation with the Ministry of Health regarding safe, legally appropriate, protocol-based pathways for expanding on-site treatment capacity under physician oversight.

Finally, advance care planning should be integrated into routine communication with families and legal proxies. Where clinically and legally appropriate, hospitalization preferences and do-not-hospitalize decisions should be discussed and documented before acute deterioration occurs. Strengthening palliative-care support and symptom-management options may help align transfer decisions with residents’ goals of care while preserving continuity of care and resident safety.

### 4.2. Limitations

This study was conducted in a single dementia-specialist long-term care facility with specific staffing, physician-coverage, diagnostic-capacity, regulatory, and referral-pathway characteristics. Therefore, transferability to smaller, non-specialist, or differently resourced long-term care facilities should be interpreted cautiously. The interview sample included mainly nursing staff and only two physicians, reflecting the actual physician staffing of the participating units during the study period. Nevertheless, this limits the breadth of medical perspectives represented, and allied health perspectives were not included.

The retrospective data described referral outcomes but did not allow assessment of the clinical appropriateness or avoidability of individual transfers; therefore, non-hospitalization should not be interpreted as evidence that a referral was unnecessary. The retrospective referral analysis was also limited by the quality and specificity of routine documentation, including the residual “Other causes” category. Predictive modeling of hospitalization after referral was not conducted because referral events were clustered within residents and the dataset lacked sufficient resident-level clinical covariates. In addition, the study reflects staff perspectives only: residents with dementia, family members, and legal proxies were not interviewed. Interview accounts may have also been subject to recall and social desirability bias. Findings regarding family involvement and medico-legal concerns should therefore be interpreted as staff-perceived influences. Finally, the study did not include post-return outcome data, such as readmission, delirium, functional decline, pressure injuries, or changes in care needs after return to the facility.

## 5. Conclusions

This study shows that hospital transfer decisions for residents with dementia in long-term care are shaped by both clinical deterioration and the organizational context in which care is delivered. Transfer decisions were influenced by institutional diagnostic and treatment capacity, interprofessional roles, staff experience, family expectations, medicolegal concerns, and continuity-of-care considerations. These findings suggest that transfer decisions should not be understood only as individual clinical judgments, but as complex clinical–organizational decisions made under conditions of uncertainty.

Strengthening decision-making in dementia long-term care requires structured pre-transfer assessment, clear decision-support protocols, timely medical consultation, improved access to diagnostic and treatment options, and targeted support for less experienced staff. Family communication and advance care planning should also be integrated into routine care to clarify preferences before acute deterioration occurs. Preserving continuity of care should be considered a resident-safety priority when determining whether a resident can be safely monitored or treated within the facility or require transfer to hospital care.

## Figures and Tables

**Table 1:** Characteristics of Study Participants.

Characteristic	n
**Professional group**	
Nursing staff	15
Physicians	2
Total	17
**Professional experience**	
Less than 5 years	5
5–10 years	3
More than 10 years	9
**Length of employment at the facility**	
Less than 5 years	8
5–10 years	5
More than 10 years	4

*Note. Participant characteristics are presented in aggregated form to reduce the risk of deductive identification in this single-institution sample. Individual characteristics are not linked to quotation identifiers.*

**Table 2:** Referral Outcomes by Ward Type.

Ward Type	Referrals, n (%)	Hospitalization, n (%)	No Hospitalization, n (%)	Family Refused to Transfer, n (%)
Ambulatory residents with dementia	159 (27.5%)	48 (30.2%)	108 (67.9%)	3 (1.9%)
Fully dependent, non-ambulatory residents with dementia	419 (72.5%)	154 (36.8%)	262 (62.5%)	3 (0.7%)
Total	578 (100%)	202 (34.9%)	370 (64.0%)	6 (1.0%)

*Note. Percentages in the Referral events column are calculated out of all 578 referral events. Percentages for hospitalization, no hospitalization, and family refusal are calculated within each type of ward. Data are presented descriptively; no inferential comparisons between ward types were conducted because referral events may include repeated referrals involving the same resident.*

**Table 3:** Distribution of Referral Reason Categories by Ward Type.

Referral Reason Category	Ambulatory Residents with Dementia, n (%)	Fully Dependent, Non-Ambulatory Residents with Dementia, n (%)	Total, n (%)
Respiratory deterioration/pneumonia/aspiration	23 (14.5%)	95 (22.7%)	118 (20.4%)
Fall, trauma, head injury, or suspected fracture	50 (31.4%)	43 (10.3%)	93 (16.1%)
Suspected DVT/limb swelling	13 (8.2%)	36 (8.6%)	49 (8.5%)
Infection, sepsis, fever, or UTI	14 (8.8%)	34 (8.1%)	48 (8.3%)
Gastrointestinal symptoms or bleeding	9 (5.7%)	35 (8.4%)	44 (7.6%)
Neurological events or altered consciousness	12 (7.5%)	22 (5.3%)	34 (5.9%)
Cardiovascular instability	4 (2.5%)	21 (5.0%)	25 (4.3%)
Skin/eye conditions	4 (2.5%)	12 (2.9%)	16 (2.8%)
Urological/gynecological issues	2 (1.3%)	9 (2.1%)	11 (1.9%)
Other causes	28 (17.6%)	112 (26.8%)	140 (24.2%)
Total	159 (100%)	419 (100%)	578 (100%)

*Note. Percentages are calculated within each ward type and for the total sample of referral events. Referral reasons were grouped into clinically related categories based on the documented reason for referral. Data are descriptive only; no inferential comparisons between ward types were conducted. DVT = deep vein thrombosis; UTI = urinary tract infection.*

**Table 4:** Classification of main themes and subthemes.

Main Themes	Subthemes
Theme 1: “*From recognition to authorization*”: Interprofessional hierarchy in referral decision-making	*Initial nursing assessment and identification of clinical change*: Nurses were the first to identify changes in residents’ conditions, assess deterioration, and report their findings to physicians. *Hierarchy of authority and professional safety nets*: Physicians held formal authority to approve transfer, whereas consultation with colleagues, senior nurses, nursing managers, and physicians helped reduce uncertainty, strengthen confidence, and share responsibility.
Theme 2: “*When uncertainty becomes risk*”: Clinical uncertainty, limited resources, and defensive transfer	*Decision-making under limited information and resource constraints*: Referral decisions were made amid incomplete information, time pressure, limited diagnostic capacity, and restricted treatment options within the facility. *Hospital transfer as a defensive and systemic risk management strategy*: In uncertain situations, transfer became the safest and most defensible option for managing clinical, professional, legal, and family-related risks.
Theme 3: “*Protecting continuity*”: Balancing hospital transfer against the harms of disruption	*Risk versus benefit*: *Transfer-related harms for residents with dementia*: Hospital transfer was perceived as potentially harmful as it could disrupt routine and contribute to delirium, agitation, infections, pressure injuries, functional decline, and cognitive deterioration. *Professional experience and local management*: Participants’ accounts suggested that professional experience shaped how staff managed uncertainty, with less experienced staff perceived as more likely to seek hospital transfer as a risk-reducing strategy.

## Data Availability

The data supporting the findings of this study are not publicly available because they contain sensitive information derived from residents’ clinical records and staff interviews. De-identified data may be available from the corresponding author upon reasonable request, subject to ethical approval, institutional permission, and compliance with applicable data protection regulations.

## Supplementary Materials

The following supporting information can be downloaded at https://www.mdpi.com/article/10.3390/healthcare14203365/s1, Supplementary File S1: COREQ (Consolidated criteria for Reporting Qualitative research) Checklist [28].
